# Interobserver and Intraobserver Agreement of Sonographic BIRADS Lexicon in the Assessment of Breast Masses

**DOI:** 10.5812/iranjradiol.10708

**Published:** 2013-08-30

**Authors:** Eda Elverici, Betul Zengin, Ayse Nurdan Barca, Pinar Didem Yilmaz, Aysegul Alimli, Levent Araz

**Affiliations:** 1 Department of Radiology, Ankara Numune Education and Research Hospital, Ankara, Turkey; 2 Department of Radiology, Yozgat State Hospital, Yozgat, Turkey; 3 Department of Radiology, Artvin State Hospital, Artvin, Turkey

**Keywords:** Mammography, Breast, Ultrasonography

## Abstract

**Background:**

BI-RADS was first developed in 1993 for mammography and in 2003 it was redesigned for ultrasonography (US). If the observer agreement is high, the method used in the classification of lesion would be reproducible.

**Objectives:**

The aim of this study is to evaluate the inter- and intraobserver agreement of sonographic BI-RADS lexicon in the categorization and feature characterization of nonpalpable breast lesions.

**Patients and Methods:**

We included 223 patients with 245 nonpalpable breast lesions who underwent ultrasound-guided wire needle localization. Two radiologists retrospectively described each lesion using sonographic BI-RADS descriptors and final assessment. The observers were blinded to mammographic images, medical history and pathologic results. Inter- and intraobserver agreement was assessed using Kappa (κ) agreement coefficient.

**Results:**

The interobserver agreement for sonographic descriptors changed between fair and substantial. The highest agreement was detected for mass orientation (κ=0.66). The lowest agreement was found in the margin (κ=0.33). The interobserver agreement for BI-RADS final category was found as fair (κ=0.35). The intraobserver agreement for sonographic descriptors changed between substantial and almost perfect. The intraobserver agreement of BI-RADS result category was found as substantial for observer 1 (κ=0.64) and excellent for observer 2 (κ=0.83).

**Conclusion:**

Our results demonstrated that each observer was self-consistent in interpreting US BI-RADS classification, while interobserver agreement was relatively poor. Although it has been ten years since the description of sonographic BI-RADS lexicon, further training and periodic performance evaluations would probably help to achieve better agreement among radiologists.

## 1. Background

With recent developments in ultrasound equipment, sonography (US) is now a well-established tool in breast imaging, allowing identification of up to 27% of breast masses that are occult on mammography, especially in women under the age of 50. The use of assessment categories, described in BI-RADS for US, allows malignant solid masses to be distinguished from benign ones at least as accurately as mammography (1). The specificity of mammography increases with the use of US; especially the number of false negative lesions in dense breasts and the false positive lesions that could lead to biopsy is decreased (2). The sensitivity of breast sonography has been found to be superior to mammography especially in premenopausal breasts. In addition, US plays a crucial role in interventional procedures such as fine needle aspiration biopsy and preoperative localization (3).

Breast US is disadvantageous by means of lack of reproducibility regarding lesion characterization, particularly for small lesions (4). Breast US is also highly operator dependent (5). The American College of Radiology (ACR) has developed the Breast Imaging, Reporting and Data System (BI-RADS) in order to provide a common language in the classification of breast lesions and to provide a clear result for the clinician (6). BI-RADS was first developed in 1993 for mammography and in 2003 it was redesigned for US (7).

If the observer agreement is high, the method used in classification of the lesion would be reproducible (8). Therefore, various studies have been conducted in order to evaluate the inter- and intraobserver agreement of BI-RADS for mammography. There are few studies evaluating the observer agreement of BI-RADS lexicon for US, despite its introduction in 2003 (2, 4, 9, 10).

## 2. Objectives

The aim of this study is to evaluate the inter- and intraobserver agreement of sonographic BI-RADS lexicon in nonpalpable breast lesions.

## 3. Patients and Methods

### 3.1. Study Design

Approval of the ethical review board was obtained prior to the study (Approval number: 2011-147). Between January 2008 and 2011, 223 patients with 245 nonpalpable breast lesions with at least two static sonographic images obtained prior to ultrasound-guided wire needle localization for excisional biopsy, were included in the study. Sonographic features of the lesions and BI-RADS scores were retrospectively evaluated.

### 3.2. Imaging Protocol

The evaluation of the lesions was performed by ultrasound (Logic 7, General Electric Medical Systems; Milwaukee, USA) using 10-14 MHz linear probe. At least two static images of the lesions in two orthogonal positions were obtained by an experienced radiologist who was different from observers 1 and 2.

### 3.3. Evaluation of Sonographic Images

The static images were retrospectively evaluated twice by two radiologists with 10 and 14 years of experience in breast imaging. Observers waited for two months between the two assessments. The observers were blind to the clinical data, mammography images and the pathology results of each patient.

The observers evaluated the lesions using the fourth edition of the BI-RADS lexicon. The lesion shape (oval, round, irregular), orientation (parallel, non-parallel), margins (circumscribed, indistinct, angular, microlobulated, spiculated), lesion boundary (abrupt interface, echogenic halo), echo pattern (anechoic, hyperechoic, complex, hypoechoic, isoechoic) and posterior acoustic features (no posterior alteration, enhancement, shadowing, combined pattern) were evaluated. Observers chose the single, most suitable lesion descriptor for each category. BI-RADS criteria of surrounding tissue changes, calcification and vascularization were not evaluated because evaluation of these features using static images was highly difficult. The observers chose the most suitable BI-RADS category at the end of the evaluation. The lesions were classified as BI-RADS 3 (most probably benign), BI-RADS 4a (low suspicion of malignancy), BI-RADS 4b (intermediate suspicion of malignancy), BI-RADS 4c (moderate suspicion of malignancy) and BI-RADS 5 (high suspicion of malignancy).

### 3.4. Statistical Analysis

Inter- and intraobserver agreement was evaluated for lesion descriptors and the final BI-RADS category. Data analysis was carried out with SPSS for Windows Ver. 11.5 pocket program (SPSS Inc., Chicago, Illinois, USA). Descriptive statistics were shown as the number of observations and percentage. Inter- and intraobserver agreement of clinical evaluation was performed with Kappa coefficient (κ) calculation. The guidelines of Landis and Koch were followed in interpreting Kappa values: 0.00-0.20, slight agreement; 0.21-0.40, fair agreement; 0.41-0.60, moderate agreement; 0.61-0.80, substantial agreement; and 0.81-1.00, almost perfect agreement (11). For possible dichotomizations of variables and combinations of observers, agreement values were estimated with 95% confidence intervals (CIs).

## 4. Results

The mean age of the patients was 48.6 (23-77) years. All lesions were non-palpable, the mean length of their long axis was 9.6 mm (3-30 mm), and the mean length of their short axis was 5.9 mm (1.5-16 mm).

Histopathological diagnosis of 237 lesions could be obtained of which 49 (20.6%) were malignant, 43 (18.1%) were high risk lesions and 145 (61%) were benign lesions. The most common benign pathology was columnar cell lesion (CCL) (26.2%), fibrocystic changes (24.1%), ductal epithelial hyperplasia (DEH) (18.6%), and fibroadenoma (9.6%). The most common malignant lesion was invasive ductal carcinoma (63.2%), and the most common high risk lesion was atypical CCL (53.4%).

### 4.1. Interobserver Agreement

The interobserver agreement for sonographic BI-RADS lesion descriptors changed between fair and substantial. The highest agreement was detected for mass orientation (κ=0.66) (95% CI: 0.60-0.72). Furthermore, the agreement for shape, lesion boundaries, echo pattern and posterior acoustic features were found as moderate (κ=0.45, κ=0.56, κ=0.41, κ=0.54) (95% CIs: 0.41-0.49; 0.49-0.62; 0.35-0.47; 0.49-0.59). The lowest agreement was found in margin (κ=0.33) (95% CI: 0.29-0.40). The findings are summarized in Table 1. 

**Table 1.** Interobserver Agreement for Sonographic BI-RADS Descriptors

BI-RADS Descriptors	κ value
**Shape**	0.45
**Orientation**	0.66
**Margin**	0.33
**Lesion boundary**	0.56
**Echo pattern**	0.41
**Posterior acoustic features**	0.54

The interobserver agreement for BI-RADS final category was found as fair (κ=0.35) (95% CI: 0.29-0.41). For BI-RADS 3 and 4, the agreement was moderate (κ=0.42, κ=0.47) (95% CIs: 0.38-0.48; 0.40-0.54). The highest agreement was detected for BI-RADS 5 (κ=0.65) (95% CI: 0.58-0.75). The interobserver agreement for final BI-RADS category is summarized in Table 2. 

**Table 2.** Interobserver Agreement for BI-RADS Final Categories

BI-RADS Category	κ value
**Category 3**	0.42
**Category 4**	0.47
Category 4a	0.34
Category 4b	0.22
Category 4c	0.33
**Category 5**	0.65
**Overall**	0.35

### 4.2. Intraobserver Agreement

The intraobserver agreement for sonographic BI-RADS lesion descriptors changed between substantial and almost perfect for observer 1 and 2. While the intraobserver agreement for shape and orientation was found as almost perfect for observer 1 (κ=0.85, κ=0.84) (95% CIs: 0.80-0.92; 0.77-0.91); agreement for shape, orientation, margin, lesion boundary and posterior acoustic features was found as almost perfect for observer 2 (κ=0.91, κ=0.94, κ=0.83, κ=0.94, κ=0.94) (95% CIs: 0.88-0.95; 0.90-0.98; 0.75-0.92; 0.90-0.98; 0.90-0.99). The intraobserver agreement for margin, lesion boundary, echo pattern and posterior acoustic features were found as substantial for observer 1 (κ=0.71, κ=0.71, κ=0.68, κ=0.79) (95% CIs: 0.65-0.79; 0.61-0.81; 0.61-0.76; 0.70-0.88); only the echo pattern was found as substantial agreement for observer 2 (κ=0.71) (95% CI: 0.65-0.78). Intraobserver agreement details for sonographic BI-RADS descriptors are summarized in Table 3. 

**Table 3.** Intraobserver Agreement for Sonographic BI-RADS Descriptors

BI-RADS Descriptors	Observer 1 κ value	Observer 2 κ value
**Shape**	0.85	0.91
**Orientation**	0.84	0.94
**Margin**	0.71	0.83
**Lesion boundary**	0.71	0.94
**Echo pattern**	0.68	0.71
**Posterior acoustic features**	0.79	0.94

The intraobserver agreement of BI-RADS final category was found as substantial, and almost perfect for observer 1 and 2, respectively (κ=0.64, κ=0.83) (95% CIs: 0.59-0.69; 0.79-0.88). Intraobserver agreement for observer 1 was found as substantial for BI-RADS 3 and 4 (κ=0.76, κ=0.77) (95% CI: 0.70-0.83; 0.72-0.83), and almost perfect for BI-RADS 5 (κ=0.86) (95% CI: 0.81-0.91); it was found as substantial for BI-RADS 3 (κ=0.77) (95% CI: 0.72-0.82), and almost perfect for BI-RADS 4 and 5 (κ=0.94, κ=0.94) (95% CI: 0.88-0.98; 0.89-0.98) for observer 2. Intraobserver agreement for BI-RADS final categories are summarized in Table 4. 

**Table 4.** Intraobserver Agreement for BI-RADS Final Categories

BI-RADS Categories	Observer 1 κ value	Observer 2 κ value
**Category 3**	0.76	0.77
**Category 4**	0.77	0.94
Category 4a	0.59	0.82
Category 4b	0.52	0.71
Category 4c	0.67	0.90
**Category 5**	0.86	0.94
**Overall**	0.64	0.83

## 5. Discussion

With the increasing use of US for breast lesions, ACR described BI-RADS classification for US in 2003 to obtain a lingua franca and to determine a more accurate description for clinicians (7). BI-RADS classification for mammography has been proposed since 1993. While there are many studies focused on interobserver agreement for image exams of mammography, studies concerning the agreement of US BI-RADS lexicon are few. Previous studies were published relatively in the early period of US BI-RADS description. We aimed to add our experience after ten years of worldwide usage of US BI-RADS lexicon. In our study, we evaluated both intraobserver and interobserver agreements for BI-RADS US classification. Our intraobserver agreements varied between substantial and almost perfect, while interobserver agreements varied between fair and substantial. Our results are compatible with many of the studies subjected on agreement variability of BI-RADS US classification (2, 4, 9, 10, 12). Lazarus et al. (12) published the first study on interobserver agreement for BI-RADS US in 2006. In this study, interobserver agreement for the sonographic BI-RADS descriptors ranged between fair and substantial agreement, both for the evaluation of lesion features and final BI-RADS category determination. The Kappa values of interobserver variability for previous studies and our study evaluating the sonographic BI-RADS descriptors are shown in Table 5. 

**Table 5.** Interobserver Variability for Previous Studies Evaluating the Sonographic BI-RADS Descriptors

Description & Final Assessment	Our Study κ value	Lazarus et al. (12) κ value	Berg et al. (13) κ value	Park et al. (2) κ value	Lee et al. (10) κ value	Abdullah et al. (4) κ value
**Shape**	0.45	0.66	0.62	0.42	0.49	0.64
**Orientation**	0.66	0.61	0.72	0.61	0.56	0.70
**Margin**	0.33	0.40	0.67	0.32	0.33	0.36
**Lesion boundary**	0.56	0.69	0.36	0.55	0.59	0.48
**Echo pattern**	0.41	0.29	0.25	0.36	0.37	0.58
**Posterior feature**	0.54	0.40	0.38	0.53	0.49	0.47
**Final category**	0.35	0.28	0.52	0.49	0.53	0.30

In our study, the interobserver agreement in the use of sonographic BI-RADS lexicon for shape was found as moderate. This ratio was similar to the studies of Park et al. (2) and Lee et al. (10). Furthermore, in the studies conducted by Lazarus et al. (12), Abdullah et al. (4) and Berg et al. (13), the interobserver agreement for shape was found as substantial. The highest agreement for shape was found in the study carried out by Abdullah et al. (4) (κ=0.64). In this study, when the lesion dimensions are grouped to <0.7cm and >0.7cm, the interobserver agreement for small lesions (<0.7) was similar to our study (κ=0.48). In our study, all the lesions were non-palpable and their mean dimensions were smaller than 1 cm. In small lesions, sonographic descriptors such as shape and margins are especially difficult to evaluate.

In our study, the highest agreement was found for orientation that was substantial. This ratio was similar to other studies (2, 4, 12, 13). The higher agreement levels for orientation can be explained by easier description of parallel and non-parallel orientation than evaluating other features with more parameters included (2).

The highest agreement rate for lesion margins was found in the study of Lazarus et al. (12), while the lowest was detected in the study performed by Berg et al. (13). Other studies (2, 4, 10) are at moderate agreement, similar to our study.

The interobserver agreement for echo pattern in some studies was slight (2, 10, 12, 13). Abdullah et al. (4) detected the highest agreement similar to our study that was moderate. This shows that the observers had difficulty in this categorization. However, the echo features are not considered as an important criteria in predicting malignant from benign (14).

In our study, interobserver agreement for margin was found as fair, similar to other studies (2, 4, 10, 12). Margin features are of the most important parameters in choosing final BI-RADS category and making the biopsy decision; however, 5 subgroups are defined in sonographic BI-RADS lexicon for margins (circumscribed, indistinct, angular, microlobulated and spiculated). It is very difficult to choose only one of these subgroups using static images. In a prospective study conducted by Berg et al. (13), the agreement for margin was the highest (κ=0.67) compared to other studies. Because in this study, the margin descriptor had two alternatives; circumscribed and non-circumscribed. It is clear that deciding whether a lesion has a circumscribed margin or not is easier; however, choosing a subgroup for non-circumscribed is difficult. In fact, it is not a problem because one of either four descriptors should be assigned as a suspicious finding, so the final assessment would not be affected (10).

The interobserver agreement for final BI-RADS category in our study was found as fair similar to the studies performed by Lazarus et al. (12), Abdullah et al. (4) and Lai et al. (5). Based on the study conducted by Abdullah et al. (4), the low levels are due to subcategorizing of BI-RADS 4 to 4a, 4b, 4c. When category 4 was evaluated as a whole, interobserver reproducibility increased to moderate agreement (κ=0.47). In our study, the agreement for BI-RADS 4 was found as moderate, but when categorized as BI-RADS 4 a-b-c, the agreement decreased to fair. This indicates that the subcategorization of BI-RADS 4 lesions, which embrace a wide range, is not clearly defined.

In the studies carried out by Park et al. (2) and Berg et al. (13), the agreement for the final category of BI-RADS was found as moderate and these rates were higher than our study. The reason of higher final BI-RADS agreement in the study of Berg et al. (13) was the non-homogeneous distribution of patients. Of the 88 patients, 42 were categorized as BI-RADS category 1 and 2, 41 as category 3 and only 5 as category 4a, 4b, 4c and 5 which means there are very few subcategories difficult for observers to categorize in the final categorization. Evaluating the agreement levels without subcategorizing category 4 is the main reason for high final BI-RADS agreement results in the study of Park et al. (2).

In our study, despite the fair interobserver agreement for the final category of BI-RADS, it was found as substantial for BI-RADS category 5. This suggests that the observers provided consensus in predicting malignant lesions, but their opinion for possibly benign (BI-RADS 3) and suspicious lesions (BI-RADS 4) was variable.

Our study showed a higher level of intraobserver agreement than interobserver agreement. Our intraobserver agreement results are similar to the literature or better (2, 9, 10). The κ values of previous studies and our study, evaluating the intraobserver variability for sonographic BI-RADS descriptors are presented in Table 6. 

**Table 6.** Intraobserver Variability for Previous Studies Evaluating the Sonographic BI-RADS Descriptors.

Descriptors & Final Assesment	Our Study *κ* value	Park’s Study κ value (2)	Lee’s Study κ value (10)	Calas’ Study κ value (9)
**Shape**	0.85-0.91	0.73	0.56-0.72	-
**Orientation**	0.84-0.94	0.68	0.75-0.83	-
**Margin**	0.71-0.83	0.64	0.53-0.61	-
**Lesion boundary**	0.71-0.94	0.68	0.56-0.85	-
**Echo pattern**	0.68-0.71	0.65	0.67-0.81	-
**Posterior feature**	0.79-0.94	0.64	0.67-0.82	-
**Final categoery**	0.64-0.83	0.74	0.72-0.79	0.37-0.75

The intraobserver agreement in the study of Park et al. (2) was found as substantial both for the lesion descriptors and final BI-RADS category. In the study of Lee et al. (10), intraobserver agreement for lesion descriptors varied from moderate to almost perfect, and for the final BI-RADS category, the agreement was substantial. In the study performed by Calas et al. (9), only the final BI-RADS category was evaluated that was fair to substantial.

Our study had limitations. First, BI-RADS category 2 and 3 lesions were excluded from the study because only patients who underwent excisional biopsy after guide wire localization were included. Because the radiologists knew that only patients undergoing biopsy were included in the study, they tried to evaluate the lesions more cautiously. Second, observers only evaluated static images of the lesions, but routinely, real time US evaluation was performed. Third, the study was based on the performance of experienced radiologists on breast sonography. Inconsistencies and errors in using BI-RADS terminology among our observers may be a causative factor for a lower level of interobserver agreement than intraobserver agreement.

In conclusion, our results demonstrated that each observer was self-consistent in interpreting US BI-RADS classification, while interobserver agreement was relatively poor. Although it has been ten years since the description of sonographic BI-RADS lexicon, it has partially failed to provide a consensus among our observers. We think that feedback with pathological results of the lesions after their description by radiologists may improve the correct classification. In addition, further training and periodic performance evaluations would probably help to achieve better agreement among the radiologists.
